# Supplementary material for: Dietary Fatty Acid Intake and the Colonic Gut Microbiota in Humans
Source: Nutrients. 2022 Jun 29;14(13):2722. doi: 10.3390/nu14132722 (PMC9268849; doi:10.3390/nu14132722)
Supplement: Supplementary file 1 [file nutrients-14-02722-s001.zip › nutrients-1760154-supplementary.pdf]

nutrients

MDPI

Table S1. Relative abundance of bacterial family by fat intake

Type of fatty acids	Family	Relative abundance (%)		*q* value[a]
		Lower Intake	Higher Intake	
**Total Fat**	Sutterellaceae	0.85	2.56	<0.001
	Desulfovibrionaceae	1.05	1.84	0.007
	Acidaminococcaceae	0.52	0.99	0.02
	Pseudomonadaceae	0.10	0.11	0.02
	Christensenellaceae	0.53	0.25	0.049
**SFAs**	Bifidobacteriaceae	0.14	0.41	0.045
	Fusobacteriaceae	0.66	2.37	0.045
	Oscillospiraceae	3.07	1.02	0.045
	Veillonellaceae	0.38	0.65	0.045
**TrFAs**	Christensenellaceae	0.86	0.074	0.004
	Bifidobacteriaceae	0.19	0.32	0.006
	Butyricicoccaceae	0.13	0.34	0.02
**MUFAs**	Sutterellaceae	1.11	2.41	0.0007
	Fusobacteriaceae	0.82	1.98	0.03
	Peptostreptococcaceae	0.19	0.31	0.04
	Streptococcaceae	0.62	0.27	0.04
	Anaerovacaceae	0.05	0.07	0.04
	Desulfovibrionaceae	1.10	1.82	0.04
**PUFAs**	Sutterellaceae	0.84	2.66	<0.0001
	Prevotellaceae	2.30	3.93	0.002
	Desulfovibrionaceae	1.04	1.89	0.018
	Erysipelatoclostridiaceae	2.27	2.11	0.020
	Anaerovoracaceae	0.04	0.08	0.027
	Acidaminococcaceae	0.50	1.03	0.039
	Christensenellaceae	0.50	0.25	0.039
**n3-FAs**	Acidaminococcaceae	0.47	1.06	0.007
	Ruminococcaceae	6.44	12.6	0.007
	Sutterellaceae	1.12	2.45	0.048
**n6-FAs**	Sutterellaceae	0.84	2.66	<0.001
	Prevotellaceae	2.30	3.94	0.002
	Desulfovibrionaceae	1.04	1.89	0.02
	Erysipelatoclostridiaceae	2.27	2.11	0.02

	Anaerovoracaceae	0.04	0.08	0.03
	Acidaminococcaceae	0.50	1.03	0.04
	Christensenellaceae	0.50	0.25	0.04

MUFAs: monounsaturated fatty acids; PUFAs: polyunsaturated fatty acids; SFAs: saturated fatty acids; TrFAs: Trans fatty acids. [a] *q* value for the Mann-Whitney test.

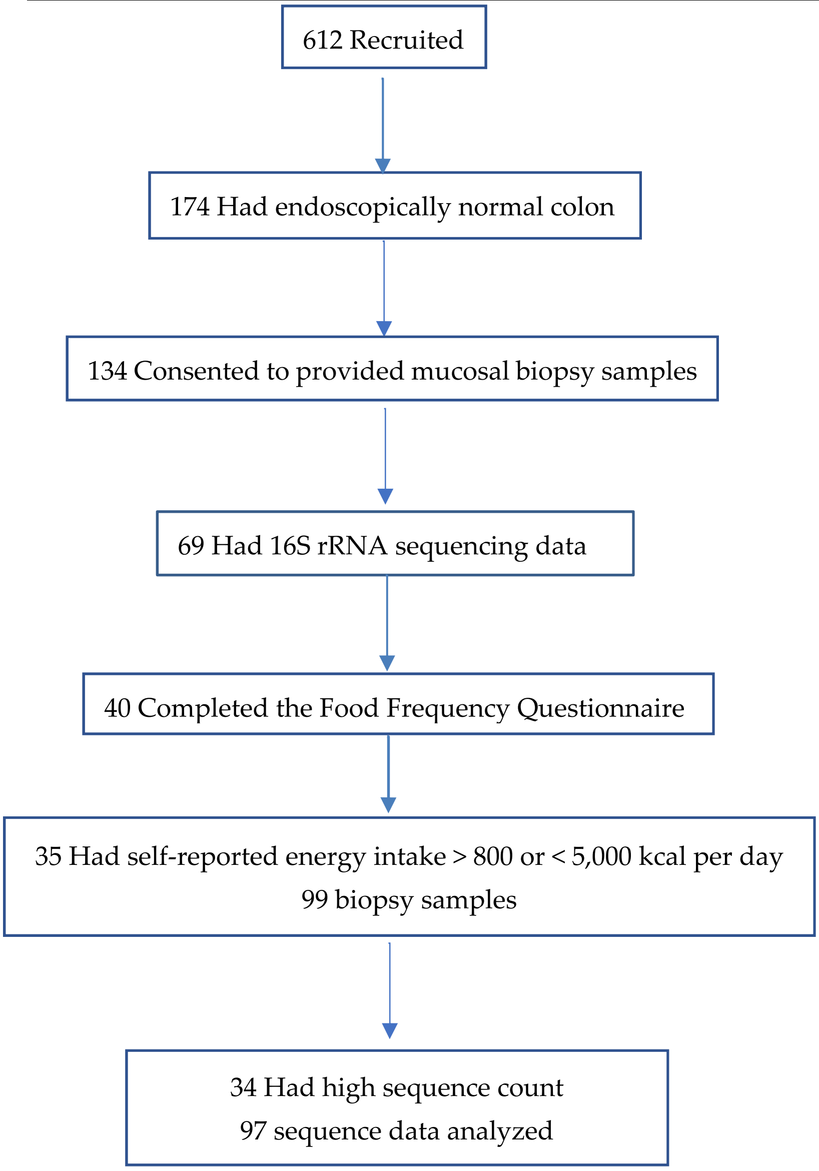

Figure S1. Flow chart of participant eligibility
